# The Circular RNA-miRNA Axis: A Special RNA Signature Regulatory Transcriptome as a Potential Biomarker for OSCC

**DOI:** 10.1016/j.omtn.2020.09.001

**Published:** 2020-09-06

**Authors:** Ramanathan Saikishore, Palanivel Velmurugan, Dhandapani Ranjithkumar, Ragunathan Latha, Thangavelu Sathiamoorthi, Ashokbabu Arun, Arumugam Veera Ravi, Subpiramaniyam Sivakumar

**Affiliations:** 1 Department of Microbiology, Science Campus, Alagappa University, Karaikudi 630003, Tamil Nadu, India; 2 Department of Biotechnology, Science Campus, Alagappa University, Karaikudi 630003, Tamil Nadu, India; 3 Department of Microbiology, Aarupadai Veedu Medical College, Puducherry 607402, India; 4 Department of Bioenvironmental Energy, College of Natural Resource and Life Science, Pusan National University, Miryang 50463, South Korea

**Keywords:** OSCC, pTNM, saliva, biomarker, circRNA-miRNA axis

## Abstract

Oral squamous cell carcinoma (OSCC) is a highly recurrent form of cancer arising from the oral epithelium, which is the result of mutational change due to etiological factors such as tobacco, smoking, chewing of areca nuts, and alcohol consumption. OSCC occurrence has been observed to be prevalent in different regions of Pacific countries and in most Asian countries. Despite the accessibility of the oral cavity, OSCC is diagnosed at an extremely late stage of pathogenic tumor node metastasis pTNM (III–IV), resulting in a poor prognosis for the individual. Therefore, it is important to make definitive, early, and efficient diagnoses. Owing to the development of omic-natured studies, the presence of proteins, transcribed elements, metabolic products, and even microflora detected in saliva helps us to select biomarkers, which is an especially exciting potential because of the availability and the non-invasive nature of sample collection. Since the discovery of circular RNA (circRNA) by Sanger sequencing, it has been reported to play a pivotal role in several human diseases, including cancer. circRNA functions as a microRNA (miRNA) sponge in the regulation of mRNA expression, forming the circRNA-miRNA regulatory axis. In the case of OSCC, overexpression of different circRNAs exhibits both tumor-progressive and tumor-suppressive effects.

## Graphical Abstract

**Figure:**
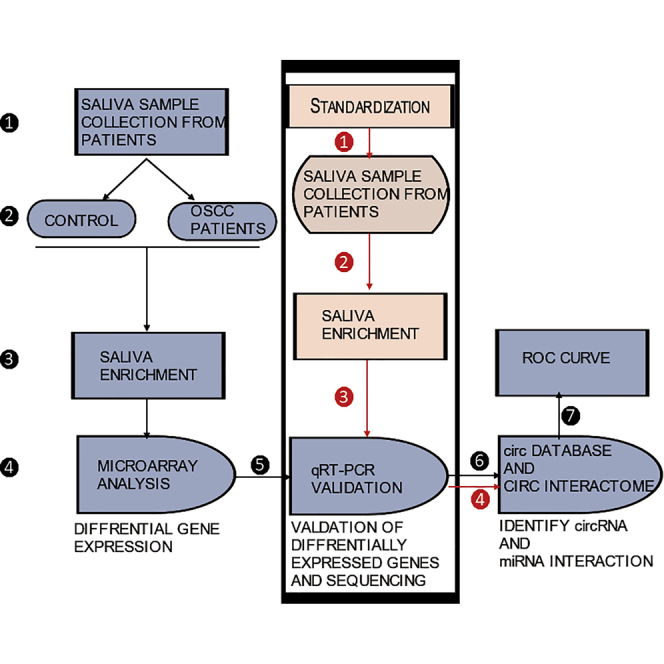

## Teaser

Oral squamous cell carcinoma (OSCC) diagnosed at an extremely late stage pTNM (III–IV) results in a poor prognosis for the individual. Hence, Velmurugan and colleagues summarize in this review omic-natured studies that use saliva components as biomarkers to diagnose OSCC at an early stage.

## Main Text

Oral squamous cell carcinoma (OSCC) is the eighth most common cancer worldwide^1^ and is also a pressing issue faced by the Indian health sector where OSCC is categorized as one among the top three cancer types in the country,^2^ commonly arising from the oral epithelium. These tend to be superficial cancers with development over the years due to many underlying factors, which were classified into four groups by WHO in 2017, including (1) the habit-associated category (major), (2) inflammatory, immune-mediated lesions, (3) lesions secondary to solar radiation, and (4) infections and rare inherited disorders (minor). These are collectively known as an oral potentially malignant disorder (OPMD), which serves as a starting point, slowly elevating to dysplasia to carcinoma *in situ*, and finally transcends to OSCC with a 5-year survival rate for fewer than 50% of the individuals.^3^

Most oral cancer (OC) is observed to transcend from leukoplakia, erythroplakia, oral lichen planus (OLP), and oral submucous fibrosis (OSMF), which are commonly seen occurring from labial mucosa, buccal mucosa, the floor of the mouth, tongue, and palate and even more aggravated due to the synergistic effects of infections from *Candida* sp. and human papillomavirus (HPV).^4^ Apart from other factors, the socioeconomic status of a population also poses as a significant risk factor for OSCC.^5^ A sociodemographic study conducted in 2013 by the Department of Public Health Dentistry, Saveetha Dental College and Hospital, Chennai, India has found that risk of OC is inversely proportional to education, income, and occupation. Similarly, most of the cases from the urban population (71.9%) and rural population (94.9%) were from the upper-lower and lower-middle class in socioeconomic scale with a very low monthly income of 5,000 rupees (Indian Rupee [INR]).^6^

Population-based cancer registered cases during 2018 reported a higher prevalence of OC to be concentrated in the central region of India compared to other surrounding regions, which was attributed to many etiological factors, with tobacco being the most important, all where the pattern of tobacco usage by the poor, uneducated population of the society was observed to be significantly high, with the majority of the population affected being the male population compared to the female population of the society.^7^ Tongue and buccal mucosa cancer are the most common types of cancer observed in the Indian subcontinent. These cancers are increasing at a rate of 0.6% (buccal mucosa cancer) and 0.35% (tongue cancer). Although tobacco and alcohol consumption rates have decreased in these younger populations, from 50% to 47% and from 38% to 34%, there has nevertheless been an increase in the incidence of OCs among the younger population.^8^ Apart from cigarettes, non-smoking tobacco, such as chewing of Areca nut wound up in betel leaf or along with tobacco in various forms, has been classified as a type 1 carcinogen and is the major factor for many cancer cases in different regions of the Pacific and most of Asia.^9^

Even though OSCCs are superficial and universally accessible, diagnosis is delayed until stage III or IV, leading to a grim prognosis for the individual.^10^ The delay in diagnoses could be due to the asymptomatic individual not showing indicative symptoms of OC, misleading the practitioner and leading to delayed diagnoses.^11^ With different stages of OSCCs, the prognosis for the individual is based on many variables that are in place, where efficient diagnoses are directly proportional to a better prognosis and aid in assigning a therapeutic program for the individuals, prolonging the 5-year survival period. A combined study performed at the Ewha Woman’s University and University of Ulsan College of Medicine at Seoul in South Korea reported higher rates of pathogenic tumor node metastasis (pTNM) in stage IV patients, with overall survival of 41.2%, reducing their 5-year survival rate compared to stage I and II, with overall survival of 93.9%.^12^ In OSCC, there is a clear need is to make an early and accurate diagnosis. Diagnosis of OSCC by using biomarkers is of enormous potential, removing bottlenecks associated with the invasive methods in the field of medicine.^13^

Using the “salivariome” as a biomarker has tremendous prospects due to its high accessibility and its non-invasive nature of specimen collection. The salivariome is an umbrella term that incorporates multiple omic-natured studies under its belt, such as (1) genomics and epigenomics, (2) proteomics, (3) transcriptomics, (4) metabolomics, and (5) microbiome-based studies.^14^ Despite their availability and accessibility, one of the major concerns with regard to biomarkers is intra-tumor heterogeneity, resulting in varying diagnostic frequency because of the branched evolution of the cancer cells where clones emerge from a common ancestor.^15^ Thus, the biomarker must cope with such a heterogenic hitch and must be universally applicable and distinct, thus facilitating the physician-designed personalized medicine therapy for the affected individual. Developing interest in circular RNA (circRNA) and recent understanding of its role in cancer by forming a regulatory axis with microRNA (miRNA) to control mRNA stability and transcription has compelled us to view this circular marvel’s expression as a potential target for a biomarker for cancer and therapy. This review discusses the prospects of the miRNA-circRNA axis as a potential biomarker for OSCC.

### Presence of circRNA and miRNA in Saliva

**Figure 1:**
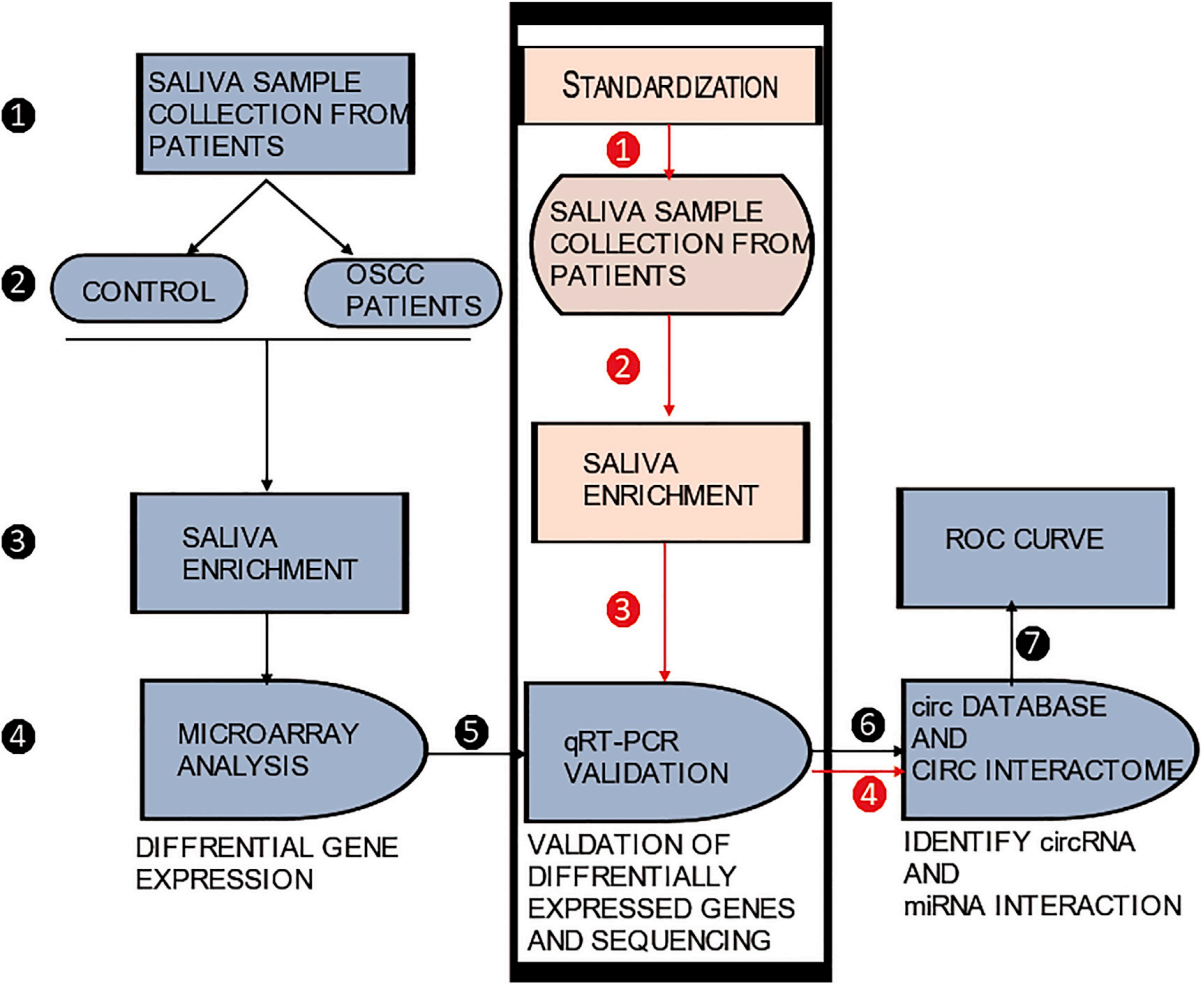
Generalized Workflow and Post-standardized Workflow Generalized workflow included the following: (1) saliva collection from (2) healthy and OSCC patients; (3) circRNA enrichment by degrading linear RNA; (4) hybridization for microarray to identify the differential regulation of circRNA between healthy and OSCC individuals; (5) validation of expression through qRT-PCR and sequencing; (6) comparison with databases to identify circRNA and miRNA interaction; (7) receiver operating characteristic (ROC) curve to know that the diagnostic value and the suitable circRNAs are standardized. Post-standardized workflow included the following: (1) after standardization, patient saliva is collected; (2) the saliva is enriched for circRNA by degrading linear RNA; (3) qRT-PCR amplification, sequencing, and comparison with reference genes; (4) comparison with databases to miRNA interaction.

Saliva displays an exciting potential for biomarker studies because of its non-invasive accessibility and abundance. A study of cell-free saliva using high-throughput RNA sequencing by Bahn et al.^16^ reported the prevalence of circRNAs in saliva and their role in intracellular signaling and the inflammatory response. These authors described that the sequenced miRNA profiles had a distinct pattern corresponding to the local cellular environment. This hints that any change in the local cellular environment might be reflected in the expression rates of miRNAs and circRNAs. Lu et al.^17^ performed microarray analysis revealing 1,850 upregulated and 1,241 downregulated lncRNAs, 10 upregulated and 24 downregulated circRNAs, and 2,729 upregulated and 883 downregulated mRNAs in the saliva of mucoepidermoid carcinoma patients. Gai et al.^18^ performed qRT-PCR on OSCC patients’ extracellular vesicle salivary samples and compared them to healthy controls, which showed upregulation of miR-412-3p, miR-512-3p, miR-302b-3p, and miR-517b-3p in OSCC. The lower expression of circRNAs and the low number of miRNA elements (MREs) predicted within the sequence pose significant challenges, but their function cannot be limited to sponging of miRNA but extends to being a scaffold to several different functions.^19^ Many more population-based studies are required to fully understand the pattern of circRNA expression in saliva, and new pipelines are being developed for understanding its more generalized function. An illustration of the generalized workflow for using saliva as diagnostic fluid is provided in Figure 1.

### circRNA Biogenesis and miRNA Sponging

Sanger et al.^20^ described a viroid via electron microscopy that revealed the absence of 5′ terminal triphosphate and degradation resistance to snake venom phosphodiesterase, discovering the presence of true closed-circular double-stranded RNA, now termed circRNA. circRNAs were previously thought to be a product of mis-splicing of premature mRNA.^21^ Distinct from the traditional canonical splicing producing linear exons by intron exclusion occurring from the 5′ downstream splice donor site to the 3′ upstream acceptor region, circRNA is a product of back-splicing from the 3′ upstream splice donor site to the 5′ downstream acceptor site, and RNA polymerase II has been reported to play a major pole in splicing events of circRNA and even in alternative circularization.^22^ Splicing factors, such as *cis*-elements, play a key role in the regulation of back-splicing of circRNAs, and the general splicing factors that usually take part in canonical splicing also help in back-splicing but with a different set of rules. Three types of splicing take place as follows: (1) lariat-driven back-splicing is regulated by first prioritizing canonical back-splicing, producing linear exons that, in turn, produce lariat intermediate-forming 3′/5′ lariat circRNAs; (2) direct back-splicing takes place when *trans*-factors or *cis*-acting elements such as RNA-binding protein (RBP), including muscle-binding proteins (e.g., MBL), quaking (QKI), and Alu repeats, which bring the downstream acceptor site to the upstream donor region, complementing intron pairing to overcome unfavorable back-splicing conditions for the spliceosome; and (3) intron lariat formation escaping debranching, producing intronic circRNA.^23^^,^^24^

**Figure 2:**
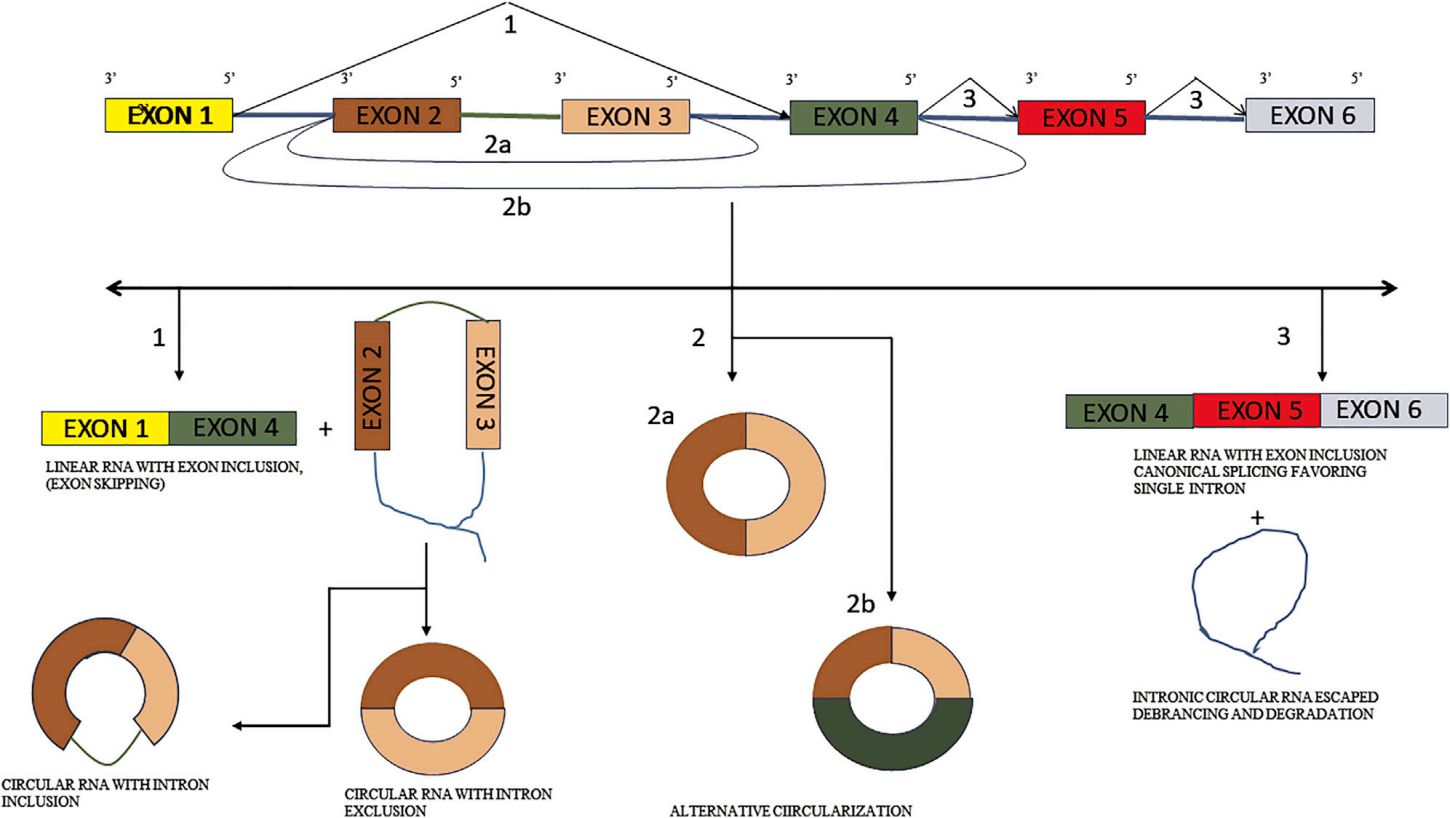
Canonical an Non Canonical Splicing of mRNA (1) Canonical splicing producing linear mRNA and intermediate lariat formation leading to back-splicing, resulting in circRNA with and without intron. (2a) Direct back-splicing regulated by RBP and (2b) alternative circularization. (3) Individual intron competition with intron lariat exclusion escaping debranching and formation of intronic circRNA.

**Figure 3:**
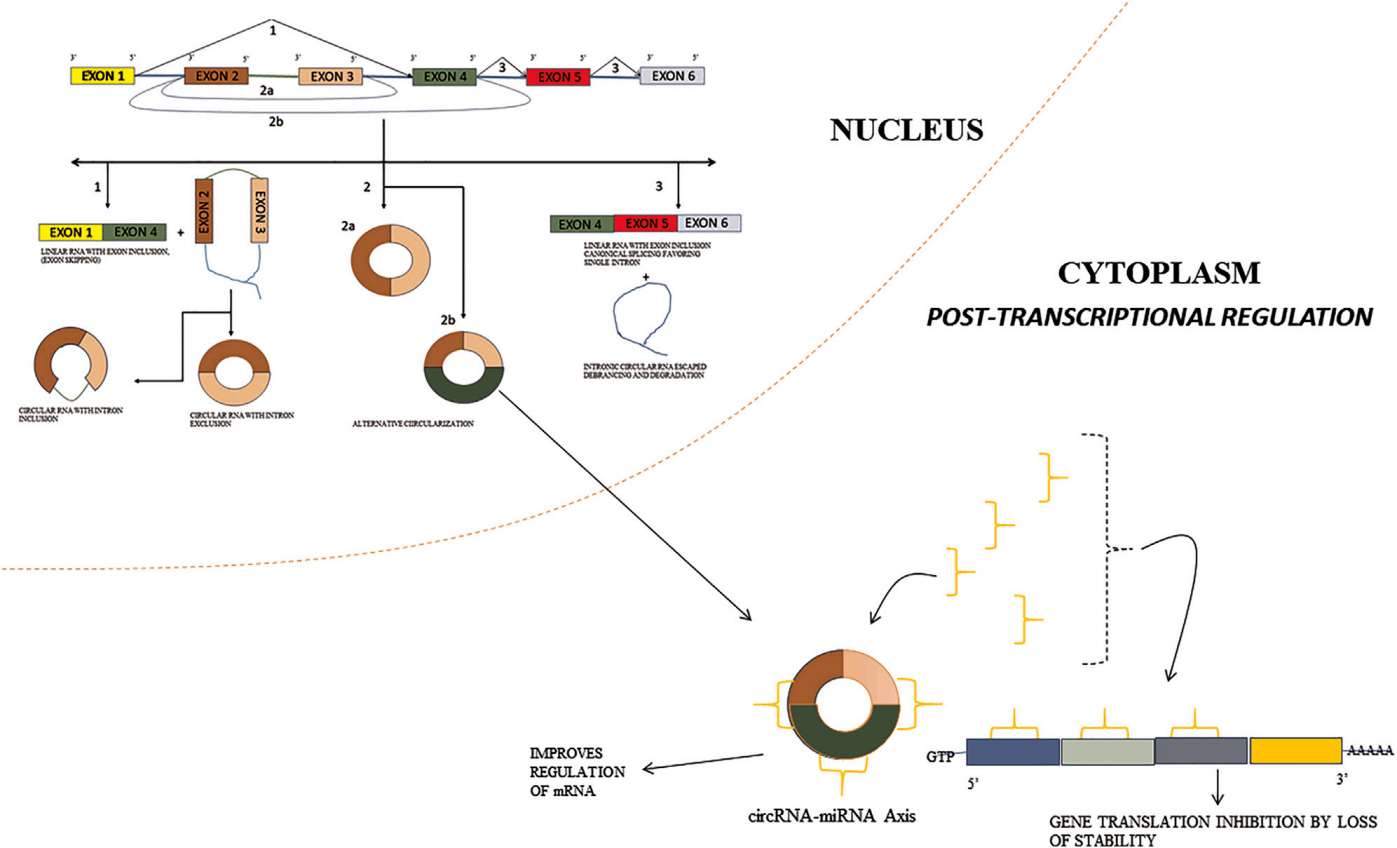
miRNA Sponge Activity by circRNA in the Cytoplasm

Alternative circularization is also observed by competition in back-splicing due to factors such as Alu repeats and produces multiple exon transcripts from a single sequence.^25^ The different back-splicing strategy is illustrated in Figure 2. The variation between the isoforms of circRNA in different tissues has also been observed.^26^ This circRNA regulates the functions of miRNAs, which are ∼25 nt long and play a role in the stability of mRNA in the cytoplasm. These miRNAs regulate cell homeostasis and also contribute to differentiation and cell development.^27^ These miRNAs might function as both tumor progressors and tumor factors, and hence the viewpoint of the specific type of disease must be taken into account.^28^ The circRNA serves as a sponge for miRNAs, disrupting their function as displayed in Figure 3, thereby promoting regulation of mRNA, forming the circRNA-miRNA axis. Their roles in OSCCs are described below.

### The miRNA-circRNA Axis Reported in OSCC

Not all circRNAs in OSCC are reported below. The only circRNA forming the circRNA-mRNA regulatory axis is described, along with a description of the role in other forms of cancer to understand the diversity of function. Table 1 provides a succinct description of the regulatory network and expression levels in OSCC tissues.

**Table 1:** A Short display of the above described circRNA-miRNA pathway regulatory axis

circRNA	Expression IN OSCC	Sponged miRNA	Regulatory Axis	Reference
ciRS-7	Upregulated	miR-671-5p	ciRS-7**/** miR-671-5p/CDR1/ AKT/ERK_1/2_/mTOR/ROS	^35^
circPVT1	Upregulated	miRNA-125b	circPVT1/ miRNA-125b /STAT3	^42^
circHIPK3	Upregulated	miR-124	circHIPK3/miR-124/ITGB1	^48^
circUHRF1	Upregulated	miR-526b-5p	circUHRF1/miR-526b-5p/c-Myc/ESRP1/TGF-β1	^50^
circMDM2	Upregulated	miR-532-3p	circMDM2/ miR-532-3p/ HK2	^53^
circPKD2	Downregulated	miR-204-3p	circPKD2/miR-204-3p/ APC2/WNT/β-catenin/p-AKT/p-ERK1/2	^59^
circDOCK1	Upregulated	miR-196a-5p	circDOCK1/miR-196a-5p/BIRC3	^63^
circATRNL1	Downregulated	miR-23a-3p	circALTR1/miR-23a-3p/PTEN/AKT/P13K/ATM/ATR/P53	^65^
hsa_circRNA_100533	Downregulated	miR-933	hsa_circRNA_100533/miR-933/GNAS	^68^
hsa_circRNA_100290	Upregulated	miR-378a	hsa_circRNA_100290/ miR-378a/GLUT1	^70^
hsa_circ_000140	Downregulated	miR-31	hsa_circ_000140/miR-31/LATS2	^72^
hsa_circ_0001971	Upregulated	miR-104, miR-204	hsa_circ_0001971/miR-104/miR-204/ P13K/AKT/FoxO3a	^74^
hsa_circ_0008309	NC: Variation but significantly showed downregulation	miR-136-5P, miR-382-5P	hsa_circ_0008309/miR-136-5P/miR-382-5P/ATX1	^77^
hsa_circ_0001162	Upregulated	miR-124	circMMP9/miR-124/AUF1	^84^

NC, not consistent

### ciRS-7

ciRS-7 is considered a pro-cancer regulator and has been shown to have 70 binding sites for miR-7.^43^ miR-7 is a key negative regulator for many cancer pathways and is a protective player in Parkinson’s disease (by downregulating α-synuclein protein).^44^ It was observed to be significantly downregulated by ciRS-7, a naturally occurring RNA circle acting as a sponge for miR-7 by regulating miR-7 knockout function, and their co-expression revealed that the expression of ciRS-7 coincided with miR-7, reducing its activity by 2-fold in the brain. Additionally, ciRS-7 was reported for the first time by Hansen et al.^45^ as a crucial candidate for influencing neurological function and brain tumor formation. ciRS-7 was also reported to be overexpressed in colorectal cancer by Weng et al.,^46^ promoting aggressiveness by downregulating miR-7 by sponging activity and interfering in the suppression of the epidermal growth factor receptor (EGFR)/RAF1/mitogen-activated protein kinase (MAPK) pathway and increasing the probability for metastasis. ciRS-7 has also been reported in non-small-cell lung cancer (NSCLC) and gastric cancer (GC).^47^ ciRS-7 is also referred to as cerebellar degeneration-related protein 1 antisense RNA (CDR1as),^48^ as it was demonstrated by Gao et al.^29^ to enhance the survival of OSCC solid tissue by inducing hypoxia-mediated autophagy, thereby enabling the cells to use autophagy as their survival mechanism to the hypoxic environment by the AKT/extracellular signal-regulated kinase (ERK)1/2/mammalian target of rapamycin (mTOR)/reactive oxygen species (ROS) pathways. Also, sponging activity forming a CDR1-miR-671-5p axis was observed to be a key promotor of autophagy in OSCC.

### circPVT1

circPVT1 is a product of the PVT1 (plasmacytoma variant translocation 1) gene locus, which was described to contain exon 3 with two flanking introns of lncPVT1^49^ in a circularized form and harboring multiple *Alu* repeats. It was first reported in gastric carcinoma by Chen et al.,^50^ having shown sponging activity toward miR-125. miR-125b has been shown to downregulate HER2 and can repress proliferation, invasion, and migration abilities in GC.^51^ circPVT1 plays a pivotal role in physiological and pathological functions, and its overexpression is related to esophageal carcinoma.^52^ The sponging activity of the cirPVT1 gene toward miRNA-497-5p was demonstrated by Verduci et al.,^53^ having resulted in upregulation of AURKA, MKI-67, and BUB1, related to cell proliferation, which are all part of the TP53 mutation, leading to the disruption of the Hippo tumor suppressor pathway where TEAD-associated factor binding induces YAP-mediated gene expression,^54^ resulting in head and neck oral squamous cell carcinoma (HNSCC). circPVT1 was observed to be significantly increased in OSCC tissues by He et al.,^30^ and it was positively correlated with tumor size, node, metastasis and displayed miRNA-125b sponging activity, resulting in upregulation of the STAT3 gene related to proliferation, thus resulting in uncontrolled cell proliferation in OSCC.

### circHIPK3

circHIPK3 has been reported to be overexpressed in lung cancer (LC) by forming a circHIPK3/miR-533-3p/SOX4/COL1A1 axis, resulting in fibroblast-to-myofibroblast transition (FMT) and fibroblast proliferation.^55^ In GC, targeting miR-107 resulted in upregulated levels of brain-derived neurotrophic factor (BDNF), leading to migration and proliferation of GC cells,^56^ colorectal cancers by forming the circHIPK3/mir-637/STAT3 axis, resulting in reduced autophagic death in cells and increasing oxaliplatin (OXA) resistance,^57^ and bladder cancer (BC), where circHIPK3 regulation is downregulated in BC and forms an axis with miR-533, reducing aggressiveness and metastasis of BC.^58^ circHIPK3 is the product of exon 2 of the HIPK3 gene.^59^ The role of circHIPK3 was first described in OSCC by Wang et al.,^31^ where circHIPK3 was upregulated in CAL 27 and SSC15 cell lines when compared with the HOK cell line, and it displayed axis formation with miR-124, resulting in progression of the proliferation of tissue in OSCC. The knockout of circHIPK3 has been shown to reverse these effects, and it upregulated miR-124. miR-124 has been shown to display tumor-suppressive activities in OSCC,^60^ where it binds to the ITGB1 gene, which is responsible for the inhibition of apoptosis, adhesion, migration, and invasion of cancer cells, where miR-124 binding to the ITGB1 gene induces apoptosis in cells and inhibits migration and invasion of tumor cells.

### circUHRF1

Zhao et al.^32^ reported a novel circRNA that is a product of exon 12 and exon 13 of the UHRF1 gene with a 301-bp length and produced by rapid circularization due to the splicing factor ESRP1 transcribed by the transcription factor c-Myc, where ESRP1 shared a GGT-rich region over the flanking introns of premature circUHRF1. c-*myc* also activates transforming growth factor (TGF)-β1, which activates TGF-β1/SMAD signaling, resulting in epithelial-to-mesenchymal transition (EMT) of OSCC. They also observed ectopic overexpression of circUHRF1, which displayed sponging activity toward miR-526b-5p, which targets the c-Myc transcription factor to regulate EMT progression, forming the circUHRF1/miR-526b-5p/c-Myc/ESRP1/TGF-β1 axis feedback loop. Overexpression of circUHRF1 often resulted in a poor prognosis for OSCC. c-Myc regulates a wide array of genes that regulate metabolism and proliferation of cells, and its expression is tightly regulated and exceptionally low due to its potential to inhibit genes with apoptotic properties.^61^ Aberrant ESRP1 expression displays proliferation and metastasis by switching from mesenchymal to epithelial phenotypes through upregulated CDHI in ovarian cancer.^62^

### circMDM2

Zheng et al.^33^ reported that overexpression of circMDM2 promoted proliferation in OSCC and played a role in glucose absorption, ATP level, and lactate production. HK2 was also observed to be upregulated with circMDM2, where HK2 is targeted by miR-532-3p. circMDM2 shows sponging activity toward miR-532-3p, promoting proliferation and glycolysis by the activation of HK2, forming a circMDM2/miR-532-3p/HK2 axis in OSCC. To fulfill the anabolic activities of tumor cells, HK2 diverts glucose into various pathways,^63^ and this helps us to view a positive correlation of circMDM2 with HK2. miR-532-3p has been reported to suppress tumor progression in colorectal cancer-inducing apoptosis by the miR-532-3p/ETS1/TGM2/Wnt/β-catenin axis,^64^ as well as tongue squamous cell carcinoma by forming miR-532-3p/CCR7/CCR21 and by preventing EMT progression, activating CCR21 due to overexpression of CCR7.^65^

### circPKD2

miR-204-3p has been reported to have tumor-suppressive effects in malignant melanoma by targeting the PAX2 gene, and in BC it acts by downregulating lactate dehydrogenase (LDH) by depleting lactate production.^66^^,^^67^ However, its role in OSCC is different. By targeting the APC2 gene promoting tumorigenesis, which is a sponging target for circPKD2 (also known as hsa_circ_0070401, a novel circRNA first reported in OSCC by Gao et al.^34^), when circPKD2 expression was dysregulated, this resulted in upregulated levels of miR-204-3p in OSCC. Overexpression of WNT signaling control plays a major role in the progression of the tumor in ovarian granulosa cell tumor and fertility.^68^ Also, aberrant WNT signaling due to downstream EGFR regulating nuclear translocation of β-catenin was also seen in OSCC, and the stability of β-catenin is influenced and disrupted by the APC2 gene, which has a β-catenin binding site, thus regulating the WNT/β-catenin pathway.^69^ Upregulated levels of miR-204-3p targeted the APC2 gene and resulted in increased β-catenin, phosphorylated (p-)AKT, and p-ERK1/2 expression, causing tumor progression, whereas overexpression of circPKD2 was demonstrated to reverse the inhibitory effects of miR-204-3p on the APC2 gene in OSCC, thus suppressing tumor progression.^34^

### circDOCK1 (hsa_circ_100721)

circDOCK1 was reported to be upregulated in BC by forming the circDOCK1/miR-132-3p/SOX5 axis, and knockdown of circDOCK1 inhibited cell proliferation and migration in Ej-m3 and 5637 cell lines.^70^ The role of circDOCK1 in OSCC was first observed by Wang et al.^35^, who observed upregulated levels of circDOCK1 in OSCC cell lines showing sponging of miR-196a-5p, which resulted in upregulated levels of BIRC3, thereby reducing the apoptotic activity, whereas silencing of circDOCK1 increased the apoptotic activity of OSCC cells. The BIRC3 gene inhibition effect has been demonstrated in liver cancer, which has been shown to reduce cell proliferation and metastasis.^71^

### circATRNL1

Chen et al.^36^ reported that the level of expression of circALTRN1 in OSCC contributed to the sensitivity to irradiation therapy in OSCC patients. These authors observed the downregulation of circALTRN1 in OSCC cells and related it to poor prognosis in patients, and the cells displayed irradiation resistance due to the upregulated levels of miR-23a-3p. miR-23a-3p inhibited cell apoptosis and G_2_ cell arrest promoting proliferation by binding to PTEN. The PTEN levels were observed to be positively correlated with the overexpression of circALTRN1 due to the sponging activity toward miR-23a-3p and improved radiosensitivity by arresting colony formation, which induced apoptosis and negatively regulated the AKT/phosphatidylinositol 3-kinase (P13K) pathway, further improving radiation sensitivity. Irradiation therapy induces ATM/ATR activation by sensing DNA damage and induces cell cycle arrest by activation of tumor suppressor p53. The relationship between ATM/ATR and PTEN has not been established, but it was observed that PTEN formed a complex with p53, protecting it from degradation, and this helps to make out a circALTR1/miR-23a-3p/PTEN/AKT/P13K/ATM/ATR/p53 regulatory axis.

### hsa_circRNA_100533

circRNA not only has a role in tumor progression but also in tumor suppression.^72^ Zhou et al.^73^ demonstrated the role of miR-933 in the progression of breast cancer by knocking out SMAD2. Zhu et al.^37^ observed that hsa_circRNA_100533 was downregulated in OSCC cell lines, especially in CAL-9 and SSC-2 cells, which promoted proliferation, migration, and inhibition of apoptosis of the cells compared to the NOK cell line, where higher expression levels were observed. hsa_circRNA_100533 was also demonstrated to form an axis, or sponging activity, with miR-933, thereby increasing apoptosis and inhibiting proliferation of the cells, but the effects were noted to be reversed by the induction of small interfering RNA (siRNA) GNAS and vice versa, linking the regulation of hsa_circRNA_100533 and miR-933 by GNAS.

### hsa_circRNA_100290

Overexpression of GLUT1 has been noted in multiple cancers and is regulated by the PI3K/AKT pathway, but based on the type of cancer, they are also observed to be downregulated.^74^ Overexpression of GLUT1 and increased co-expression of CDK6 with hsa_circ_100290 were reported in OSCC cells by Chen et al.,^38^ and the sponging activity of upregulated hsa_circRNA_100290 toward miR-378a was reported to inhibit miR-378a-mediated GLUT1 suppression, and increased CDK6 promoted further cell proliferation in OSCC cells.

### hsa_circ_000140

miR-31 has previously been reported to target SIRT3, resulting in mitochondrial activity and inhibiting ACOX1, thereby increasing motility of the cells.^75^ LATS2, a core kinase of the Hippo tumor-suppressing signaling pathway, was shown to be underexpressed in OSCC cells and a direct target of miR-31. Peng et al.^39^ observed the dysregulation of hsa_circ_000140 and related this to an increased risk of TNM stage and lymph node metastasis (LNM). They also demonstrated that knockdown of miR-31 by overexpression of circRNA_000140 sponged miR-31 and resulted in increased levels of LATS2, ultimately resulting in an increased level of YAP1 phosphorylation and inhibiting tumor progression in OSCC.

### hsa_circ_0001971

When hsa_circ_0001971 was first described by Zhao et al.^76^ with multiple miRNA target sites, such as miR-152-5p, miR-103a-3p, miR-107, miR-505-3p, and miR-9-5p, the authors hinted that the dysregulation of miR-107 and miR-103a-3p resulted in the development and progression of tumors but did not explain the role of circRNA in OSCC. Overexpression of hsa_circ_001971 in OSCC reported by Tan et al.^40^ related to a poor prognosis for the individual and by experimental knockdown inhibited malignant activities and also contributed to DDP (cisplatin) sensitivity in cell lines CAL-29 and SCC-9. miR-194 and miR-204 are potential targets for hsa_circ_0001971. miR-194 was reported as an anti-onco-miRNA by P13K/AKT/FoxO3a signaling through repression of acylglycerol kinase (AGK) expression and thus inhibiting cell proliferation.^77^ miR-204 was reported to disrupt the EMT properties of OSCC by miR-204-mediated Sox4 and Slug inhibition.^78^ The sponging activity of circ_0001971 results in downregulation of miR-194 and miR-204, resulting in inactivation of the P13K/AKT/FoxO3a pathway and in metastasis and cell growth, and the knockdown of circ_00019171 had a reverse effect and was shown to regulate OSCC.^40^

### hsa_circ_0008309

Li et al.^41^ reported the role of hsa_circ_0008309 in OSCC. Of the 45 OSCC samples, they found various types of regulation of circ_0008309, but a substantial number of samples showed downregulation, and further analysis revealed the sponging activity toward miR-136-5P and miR-382-5P, which showed interaction with ATXN1, which is involved in the Notch signaling pathway for migration of cells, but its expression varies accordingly to other types of cancer, and further analysis is needed to understand the function of ATXN1 as an oncogene or tumor suppressor. ATXN1 has been reported to promote tumor growth in cervical cancer.^79^ miR-136-5p has been reported to target UGTIA7 and ADH7 in lung squamous cell cancer.^80^ miR-382-5P downregulation has been related to increased levels of migration, invasion, and proliferation in glioma cells.^81^

### hsa_circ_0001162 (circMMP9)

circMPP9 has been reported to be overexpressed in osteosarcoma, forming the circMMP9/miR-1265/CHI3L1 axis and promoting cancer progression.^82^ circMMP9 has also been reported in glioblastoma multiforme cell tumorigenesis induced by eIF4A3, forming the circMMP9 axis with miR-124 and resulting in migration, proliferation, and invasion.^83^ miR-124 has been recognized to show both oncogenic and tumor-suppressive activity, and it is downregulated in HNSCC, LC, GC, and renal cell carcinoma (RCC).^84^ Xia et al.^42^ observed the upregulation of circMMP9 binding to AUF1 and miR-124, forming the circMMP9/miR-124 axis to stabilize MMP9 mRNA and thereby increasing invasion and metastasis, and they also observed that the MMP9 mRNA 3′ UTR binding region did not overlap with AUF1 and miR-124, indicating independent functioning.

### Conclusions

The discovery of circRNAs has impacted the whole of human transcriptome studies, providing new insights into their role in a range of human diseases from cancer to neurology. A rapid diagnosis is required to detect OSCC. However, discoveries of the circRNA-miRNA regulatory axis in OSCC have been made through developing microarray and sequencing platforms. The correlation between the reported and to-be-discovered circRNAs must be further elucidated by large-scale studies, which might help to superimpose the regulatory network of multiple circRNAs and might function as an effective biomarker helping clinicians to design personal therapies for OSCC patients. circRNAs have been detected in a wide range of body fluids. Saliva has enormous potential as a diagnostic fluid due to its ready availability and accessibility, and much analysis needs to be performed to understand the diversity and response to the local change in the environment. Apart from the function of the circRNA-miRNA network as a biomarker, studies addressing the expression of circRNAs after metastasis in different regions will also help to facilitate therapy. It has also been understood that circRNA influences irradiation therapy, which opens the door to new opportunities about controlling genes responsible for irradiation through circRNAs by sponging miRNAs. Engineered expression of circRNAs in OSCC to sponge the miRNAs responsible for tumor progression combined with irradiation therapy is a viable option. The changes in transcription and the genes responsible for the generation of circRNAs by mutation or any other factor that results in a variety of expression in circRNAs during cancer progression need deeper understanding.

## Author Contributions

R.S., A.A., and D.R. drafted the manuscript. P.V. and T.S. designed the research and drafted the manuscript. R.L., A.V.R., and S.S discussed and revised the manuscript. All authors read and approved the final manuscript.

## Conflicts of Interest

The authors declare no competing interests.
